# Supplementary material for: Examining the characteristics of social and behavior change communication intervention costs in low- and middle-income countries: A hedonic method approach
Source: PLoS One. 2023 Jun 15;18(6):e0287236. doi: 10.1371/journal.pone.0287236 (PMC10270606; doi:10.1371/journal.pone.0287236)
Supplement: S1 Table — (PDF) [file pone.0287236.s001.pdf]

S1 Table. Variance Inflation Factors (VIF) for Regressions Reported in Table 4

Media[1]										
Variables	(1) OLS[2]					(2) Tobit				
	Coeff.	SE Coeff.	T-value	P-value	VIF	Coeff.	SE Coeff.	T-value	P-value	VIF
Intensity of intervention (ref: Low)										
High	2.69	0.39	6.97	0.000	2.18	5.27	1.11	4.75	0.000	1.97
Medium	1.58	0.3	5.24	0.000	1.87	1.57	0.79	1.97	0.054	2.02
Health Areas (ref: HIV)										
FP/RH						-2.48	0.9	-2.75	0.008	2.99
MNCH						1.08	0.99	1.10	0.277	2.86
Malaria						-1.83	1.53	-1.20	0.237	1.82
Other						0.89	1.52	0.58	0.562	2.88
Intervention subtypes (ref: TV/Radio)										
Print	0.23	0.44	0.53	0.602	1.35	-0.77	0.94	-0.82	0.415	1.42
Mid media	1.54	0.51	3.05	0.000	2.07	0.82	0.92	0.89	0.377	2.25
SMS/phone	1.77	0.48	3.67	0.000	3.09	-0.44	0.92	-0.48	0.632	3.65
Mixed	-0.62	0.59	-1.05	0.297	1.69	-3.35	1.01	-3.33	0.002	1.57
Population (ref: General/youth)										
At-risk	1.19	0.42	2.83	0.007	2.18					
Socioeconomic status										
GNIpc										
Ln(GNIpc)	-0.52	0.24	-2.17	0.034	1.41	0.89	0.51	1.76	0.085	2.2
Ownership (ref: Public)										
NGO	0.7	0.4	1.74	0.089	1.45	2.28	1.03	2.22	0.031	1.47
Private	1.56	1.03	1.52	0.134	2.46	2.53	0.77	3.28	0.002	2.36
Other	0.71	0.35	2.02	0.049	1.54	0.96	0.72	1.33	0.188	2.02
Geographic Scope (ref: Local)										
District	-0.81	0.45	-1.81	0.077	3.69	-1.07	1.25	-0.86	0.396	5.40
National	-0.03	0.50	-0.06	0.955	4.42	0.88	1.32	0.67	0.508	5.58
Other	-1.01	0.59	-1.72	0.092	2.72	-1.89	1.48	-1.28	0.207	3.34
Constant	0.81	2.06	0.39	0.70		1.55	1.07	1.45	0.154	
Observations (Number of uni	66					66				
R-Squared[3]	0.713					0.965				
F-statistic	32.74***					7.18***				
Mean VIF	2.31					2.7				

Interpersonal Communication (IPC)										
	(3) OLS[2]					(4) Tobit				
	Coeff.	SE Coeff.	T-value	P-value	VIF	Coeff.	SE Coeff.	T-value	P-value	VIF
**Intensity of intervention (ref: Low)**										
**High**	29.2	4.7	6.21	0.000	2.27	4.14	0.7	5.89	0.000	1.56
**Medium**	12.59	3.16	3.98	0.000	2.07	1.56	0.38	4.09	0.000	1.46
**Health Areas (ref: HIV)**										
**FP/RH**	-15.15	5.66	-2.67	0.01	2.33	-2.05	0.83	-2.48	0.016	2.31
**MNCH**	-6.16	2.8	-2.21	0.031	1.94	-0.87	0.38	-2.28	0.026	1.94
**Malaria**	-24.39	7.14	-3.42	0.001	1.79	-3.7	0.95	-3.89	0.000	1.46
**Other**	-12.75	4.2	-3.04	0.003	1.79	-1.62	0.59	-2.76	0.008	1.91
**Intervention subtypes (ref: Group IPC)**										
**Individual IPC**	8.36	3.9	2.14	0.036	1.62	1.11	0.56	1.97	0.054	1.2
**Mixed IPC**	-1.49	3.34	-0.44	0.658	1.82	-0.18	0.46	-0.39	0.700	1.32
**Population (ref: General/youth)**										
**At-risk**	10.11	5.4	1.87	0.066	1.4	1.84	0.7	2.65	0.01	1.38
**Socioeconomic status**										
**GNIpc**						0.20	0.17	1.16	0.249	1.24
**Ln(GNIpc)**	3.88	1.6	2.43	0.018	1.46					
**Ownership (ref: Public)**										
**NGO**	23.87	6.76	3.53	0.001	2.97	3.2	0.95	3.37	0.001	1.66
**Private**	6.83	4.73	1.44	0.154	1.56	0.78	0.70	1.12	0.266	1.32
**Other**	7.13	3.57	2	0.05	1.92	0.88	0.5	1.76	0.083	1.45
**Geographic Scope (ref: Local)**										
**District**	8.73	3.72	2.35	0.022	2.28	1.05	0.51	2.08	0.042	2.26
**National**	-17.69	5.87	-3.01	0.004	1.27	-2.78	0.73	-3.79	0.000	1.84
**Other**	-13.26	5.77	-2.3	0.025	2.48	0.88	0.5	1.76	0.083	1.45
**Constant**	-35.47	13.94	-2.54	0.013		-0.68	0.64	-1.05	0.296	
**Observations (Number of uni**	79					79				
**R-Squared[3]**	0.629					0.772				
**F-statistic**	5.43***					7.07***				
**Mean VIF**	1.93					1.64				

Significance levels: *** *p* <0.01; ** *p* <0.05; * *p* <0.1.

Notes: The dependent variable for media/OLS is the natural logarithm of unit cost; for IPC/OLS is the linear unit cost; and percent of intervention subtype median unit cost fo

1. Media regressions include a dummy variable for Chen, two outliers in the dataset for SMS/phone.

2. Ordinary Least Squares Regression

3. R-squared for Tobit equations is the correlation coefficient of dependent variable and predicted values. [20]
