# Supplementary material for: Examining the characteristics of social and behavior change communication intervention costs in low- and middle-income countries: A hedonic method approach
Source: PLoS One. 2023 Jun 15;18(6):e0287236. doi: 10.1371/journal.pone.0287236 (PMC10270606; doi:10.1371/journal.pone.0287236)
Supplement: Attachment — Submitted filename: Response to reviewers.docx [file pone.0287236.s003.docx]

**RESPONSE TO REVIEWERS**

*Many thanks to both reviewers for their thoughtful comments. We have revised the manuscript based on their suggestions; please find below detailed responses to each point raised.*

Reviewer 1

1. The authors should provide more citations or explain in more detail if the hedonic pricing model has been previously used to study health-related topics.

*We have added further citations on hedonic pricing models, including for health-related topics. In addition, we added more information regarding why we are using the hedonic pricing model to examine SBCC costs. Basically, while most costing studies in global health have detailed, disaggregated data available to examine cost drivers, the SBCC cost data in the literature generally do not have consistent and reliable disaggregated data available. Because our final goal was to develop an SBCC costing tool (more on this in our response to question #11 below), we decided to use a pure hedonic pricing method approach.*

2. A more detailed introduction of the data source is needed as the current version is insufficient and overly simplistic.

*We have added a paragraph describing the database and citing further references.*

3. The authors should explain how they define "significant data quality issues" when setting the DataQuality variable.

*Many times, the SBCC cost data are not reported thoroughly in the literature, particularly in the studies that were published earlier. When there was a lack of clarity about the reporting of various elements – especially details about the denominator – we noted in the dataset that there was a data quality issue associated with the observation, and what the issue was. In addition, sometimes we had concerns about the data quality due to issues such as small sample sizes, as well as potentially missing information (e.g., one intervention described the steps followed in delivering the intervention, but it was not clear that one of the steps was included in the final costs obtained). We have added language about this to the paper. Note that the “Data quality” dummy variable was never statistically significant in any of the regressions.*

4. Reporting the VIF results, as mentioned in the methodology, would improve the paper.

*Thank you for the suggestion. We have added details about the (moderate) VIF results for the sample as a whole (ranging from 1.15 to 2.76, mean VIF of 1.85) and also included a supplemental table that displays the detailed regression results (including regression-specific VIFs) for the final regressions presented in Table 4.*

5. The authors should clarify why they chose 2005 and 2012 as cutoffs for the Technology variable.

*The timespan of articles included in the database ranges from 1978 to 2021. While the cutoffs for approximating periods of technology change are admittedly subjective, in examining the types of SBCC interventions implemented, we observed that the literature began to report several results for group interpersonal communication (IPC) interventions beginning in 2005, whereas there had only been one such study in the past, in 1987 - thus (arguably) a change in technology in how SBCC interventions were being delivered. Regarding the 2012 cutoff, the literature began reporting results for SMS interventions beginning in 2013, which we felt was indeed a significant change in technology. Thus, we settled on the time periods <2005, 2005-2012, and >2012. We have added some language to that effect to the paper.*

6. The authors should include the standard deviation of each variable in Table 2.

*Thank you for this suggestion; now included.*

7. Given the relatively small sample size, the authors should consider expanding the sample.

*We agree that a larger sample size would be preferable for conducting this analysis and note this as a limitation in the discussion section. Unfortunately, there are limited data available on SBCC unit costs, especially unit costs that use comparable denominators for analysis purposes. This dataset represents the universe of available SBCC estimates, as identified during the study period via a systematic review. As such, expanding the sample size is not feasible until more data become available through the peer reviewed or gray literature. We have added some emphasis on this in the paper, pairing it with the potential over-fitting issue noted below in #8.*

8. The authors should address the potential for over-fitting due to the high R-square value and many predictors.

*Thank you for your comment; we completely agree and have added this to the discussion section, pairing it with our discussion of the small sample issue (also noted above in #7).*

9. The authors should use italics when reporting p-values in the paper.

*Thank you for this suggestion; we have now italicized the “p” when reporting p-values.*

10. Do the authors have any recommendations regarding which intervention method is better, media or IPC?

*Evaluating the impact or cost-effectiveness of these interventions is beyond the scope of this paper; however, we have used these data in constructing business cases for SBCC for family planning, malaria, and nutrition (see* [Making the Business Case for Social and Behavior Change - Breakthrough ACTION and RESEARCH](https://breakthroughactionandresearch.org/business-case-sbc-fp/)*). We found that the cost-effectiveness of different types of SBCC interventions varies by context and other factors, i.e., one type is not consistently more cost-effective than another. We have added a brief sentence about this after the additional language we added discussing the SBCC costing tool (see response to #11 below).*

11. The authors could extend the discussion section to explain how their evaluation of SBCC costs could help in practice.

*We completely agree! In fact, this research underpins the Excel-based SBC costing tool we have developed that is freely available online and downloadable at: https://knowledgecommons.popcouncil.org/focus_sexual-health-repro-choice/135/). We are hoping that the tool will help stakeholders (e.g., implementing partners, donors, advocates) to assist in their planning and budgeting processes. We have added language and the citation to the discussion section around this SBC costing tool and have included the caveats noted in response #10 above, that impact needs to be considered in addition to costs when planning and budgeting for SBCC.*

I hope these comments are helpful, and I look forward to seeing the revised manuscript.

Reviewer #2:

1. Please check the manuscript language.

*We have reviewed the manuscript and made some small editing changes.*

2. Check you the formal (all the equations).

*Assuming the reviewer is asking to check the format of the equations, we have revised the equations to be clearer and more streamlined.*

3. The manuscript have a tactical sounds, but it needs to be more convenient phrases.

*Assuming the reviewer is asking for less technical language, we have made revisions to various sentences to make the manuscript more accessible.*

4. Check all the references and they maintain inside the manuscript.

*We have verified that all references have been correctly cited, including the citations added to this revision.*
